# Letter from the Editor in Chief

**DOI:** 10.19102/icrm.2025.16019

**Published:** 2025-01-15

**Authors:** Devi Nair


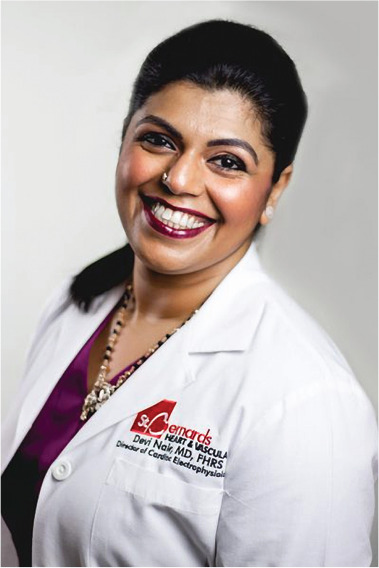


Dear Readers,

Happy New Year! As we step into 2025, I am thrilled to share highlights from the recently concluded 30th Annual AF Symposium. This landmark, international event celebrated three decades of innovation and collaboration in atrial fibrillation (AF) management. The symposium has been a cornerstone in our field, bringing together global leaders to share groundbreaking research, novel technologies, and evolving clinical practices.

## Reflections from the 30th Annual AF Symposium

This year’s program captured the essence of innovation and excellence, with a focus on the most pressing challenges and opportunities in AF management. Key highlights included:

- *Breakthroughs in ablation technologies:* Pulsed-field ablation took center stage, with new-generation systems demonstrating enhanced safety, procedural efficiency, and lesion durability.
- *Integrated stroke risk-mitigation:* Live demonstrations showcased the latest in left atrial appendage occlusion devices and their integration with ablation procedures to provide comprehensive stroke prevention. This dual-therapy approach underscores the growing importance of patient-specific, multi-modal treatment strategies.
- *Personalized care in AF management:* The symposium emphasized tailoring therapies to individual patient profiles, with sessions on the role of GLP-1 agonists in reducing AF burden and targeting epicardial adipose tissue as a modifiable risk factor.

These emerging trends highlight the shift toward addressing the root causes of AF in addition to treating the arrhythmia itself.

As we reflect on the past 30 years, it’s evident that the AF Symposium has been instrumental in driving the evolution of our field. This milestone year not only celebrated its rich history but also set the stage for the future of AF management.

## Featured in This Issue: A Year in Review

Aligned with the themes of the symposium, this month’s edition includes the article “A Year in Review: Atrial Fibrillation 2024,” authored by Drs. Amier Ahmad, Lydia Taranto, Ankur Karnik, and Rahul Doshi.^1^ This expert commentary offers a comprehensive overview of the pivotal advancements in AF management over the past year. The authors delve into the safety, efficacy, and cost considerations of pulsed-field ablation; the importance of ablation timing; and the evolving strategies for stroke prevention. Their analysis complements the discussions from the symposium, providing readers with valuable insights into the progress made in 2024 and the challenges that lie ahead.

## Looking Forward: Late-breaking Trials in February

Among the many highlights of the AF Symposium were the late-breaking clinical trials, which introduced paradigm-shifting findings in ablation, device therapy, and personalized care. In our future issues, we will feature detailed analyses and expert commentary on these pivotal studies, ensuring that our readers remain at the forefront of innovation in cardiac rhythm management.

## Embracing 2025

As we celebrate this milestone year for the AF Symposium, we are reminded of the power of collaboration and innovation in advancing our field. Our journal remains dedicated to serving as a platform for sharing transformative research, fostering dialogue, and driving progress. I look forward to another year of discovery, collaboration, and breakthroughs that will shape the future of cardiac rhythm management.

I extend my heartfelt gratitude to our authors, reviewers, and readers for their continued support. Together, we are shaping a future where our tools and therapies better serve our patients and improve lives around the world.

Wishing you a prosperous and inspiring year ahead.

Warm regards,


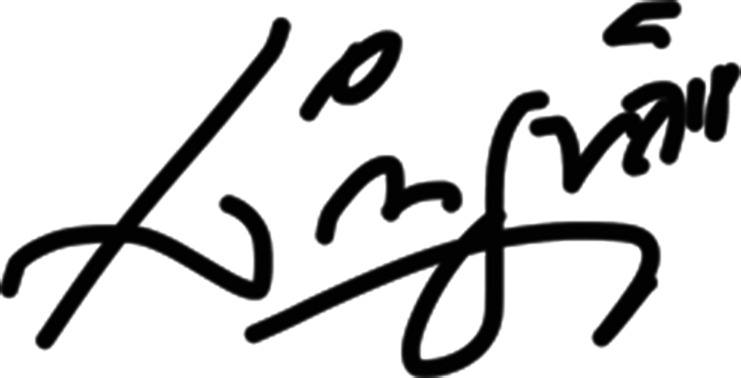


Dr. Devi Nair, md, facc, fhrs

Editor-in-Chief

*The Journal of Innovations in Cardiac Rhythm Management*

Director of the Cardiac Electrophysiology & Research,

St. Bernard’s Heart & Vascular Center, Jonesboro, AR, USA

White River Medical Center, Batesville, AR, USA

President/CEO, Arrhythmia Research Group

Clinical Adjunct Professor, University of Arkansas for Medical Sciences

Governor, Arkansas Chapter of American College of Cardiology

drdgnair@gmail.com
